# 650. Antibiotic Use among Children Seeking Medical Care with Acute Gastroenteritis within the New Vaccine Surveillance Network, United States, 2011-2021

**DOI:** 10.1093/ofid/ofad500.713

**Published:** 2023-11-27

**Authors:** Neha Balachandran, Mary Wikswo, Julie A Boom, Leila C Sahni, Rangaraj Selvarangan, Christopher J Harrison, Natasha B Halasa, Laura S Stewart, Geoffrey A Weinberg, Peter G Szilagyi, Janet A Englund, Eileen J Klein, Mary A Staat, Allison Burrell, John V Williams, Marian G Michaels, Umesh D Parashar, Sara Mirza

**Affiliations:** Cherokee Nation Assurance, Arlington, VA/ Centers for Disease Control and Prevention, Atlanta, Georgia; Centers for Disease Control and Prevention, Atlanta, Georgia; Texas Children’s Hospital, Houston, Texas; Baylor College of Medicine and Texas Children’s Hospital, Houston, Texas; Children’s Mercy Kansas City, Kansas City, Missouri; Children's Mercy Hospital, Kansas City, Missouri; Vanderbilt University Medical Center, Nashville, Tennessee; Vanderbilt University Medical Center, Nashville, Tennessee; University of Rochester School of Medicine & Dentistry, Rochester, NY; UCLA School of Medicine, Agoura Hills, California; Seattle Children’s Hospital, Seattle, Washington; University of Washington School of Medicine, Seattle, Washington; Cincinnati Children’s Hospital Medical Center, Cincinnati, Ohio; Cincinnati Children's Hospital and Medical Center, Cincinnati, Ohio; University of Pittsburgh, Pittsburgh, Pennsylvania; UPMC Children's Hospital of Pittsburgh, Pittsburgh, Pennsylvania; Division of Viral Diseases, National Center for Immunization and Respiratory Diseases, CDC, Atlanta, Georgia; Centers for Disease Control and Prevention, Atlanta, Georgia

## Abstract

**Background:**

Inappropriate antibiotic prescribing continues to be an issue in the United States (US) despite a decline in prescription rate overall. Acute gastroenteritis (AGE) often requires medical attention but is generally managed conservatively. We describe antibiotic use among children with AGE in two healthcare settings.

**Methods:**

Children aged < 5 years with AGE were enrolled from December 2011-August 2021 at 7 New Vaccine Surveillance Network (NVSN) sites across the US. Inpatients (IP) and emergency department (ED) patients with diarrhea (≥3 episodes) and/or vomiting (≥1 episode) within 24 hours, without history of noninfectious diarrhea or immunodeficiency were included. Stools were collected within 10 days of symptom onset and tested for enteric pathogens by validated molecular methods. Antibiotic use during AGE visit was collected via medical chart abstraction; antibiotic use was mostly empiric and not driven by test results. Children < 2 years with *Clostridioides difficile* detections were excluded due to high colonization rates in this group. A 20-point modified Vesikari score was calculated to categorize AGE severity [mild (0-6), moderate (7-10) and severe ( >11)].

**Results:**

Among 21,194 enrolled children [3,811 (18%) IP and 17,383 (82%) ED], median IP age was 14 months (IQR 5-28) and ED age 18 months (IQR 9-32). Most were male (IP and ED=54%) and white (IP=61% and ED=43%). Antibiotic use for AGE was noted in 994 (26%) IP and 765 (4%) ED cases; the majority were infants [IP=533 (54%), ED=295 (39%)] and had severe AGE [IP=683/901 (76%), ED=330/627 (53%)]. Fever was more common among antibiotic recipients versus non-recipients [IP=80% vs 59%, ED=81% vs 57 %]. Antibiotic use across study years was low in ED (2-10%) but higher in IP (19-31%) (Fig 1). Among tested antibiotic recipients, the predominant pathogens detected were norovirus [IP=58/422 (14%), ED=40/304 (13%)] and rotavirus [IP=89/886 (10%), ED=40/531 (8%)] without bacterial codetection, and *Enteropathogenic E. coli* [IP=10/93 (11%), ED=4/39 (10%)].

**Figure:**
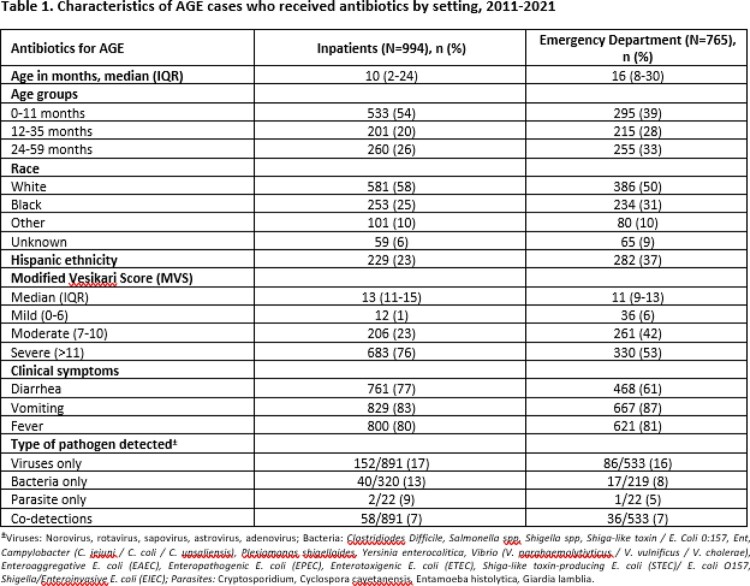

**Figure:**
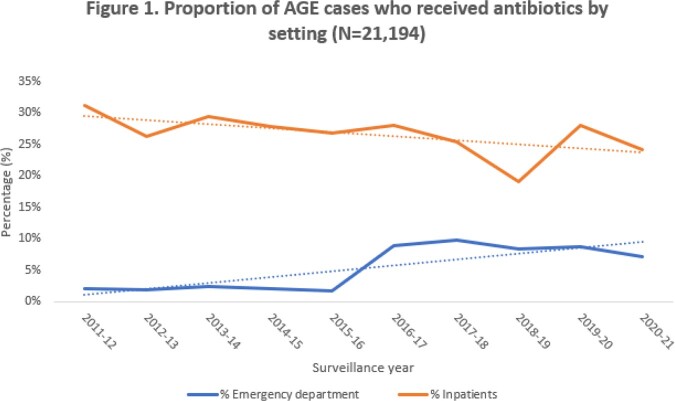

**Conclusion:**

Overall antibiotic use for AGE among enrolled children was low but was higher among IP, especially in infants and those with severe AGE. Antibiotic use for viral AGE in both ED and IP settings highlights the need to improve antibiotic stewardship among children with AGE.

**Disclosures:**

**Rangaraj Selvarangan, BVSc, PhD, D(ABMM), FIDSA, FAAM**, Abbott: Honoraria|Altona Diagnostics: Grant/Research Support|Baebies Inc: Advisor/Consultant|BioMerieux: Advisor/Consultant|BioMerieux: Grant/Research Support|Bio-Rad: Grant/Research Support|Cepheid: Grant/Research Support|GSK: Advisor/Consultant|Hologic: Grant/Research Support|Lab Simply: Advisor/Consultant|Luminex: Grant/Research Support **Christopher J. Harrison, MD**, GSK: Grant/Research Support|Pfizer: Grant/Research Support **Natasha B. Halasa, MD, MPH**, Merck: Grant/Research Support|Quidell: Grant/Research Support|Quidell: donation of kits|Sanofi: Grant/Research Support|Sanofi: vaccine support **Geoffrey A. Weinberg, MD**, Merck & Co: Honoraria **Janet A. Englund, MD**, Ark Biopharma: Advisor/Consultant|AstraZeneca: Advisor/Consultant|AstraZeneca: Grant/Research Support|GlaxoSmithKline: Grant/Research Support|Meissa Vaccines: Advisor/Consultant|Merck: Grant/Research Support|Moderna: Advisor/Consultant|Moderna: Grant/Research Support|Pfizer: Advisor/Consultant|Pfizer: Grant/Research Support|Sanofi Pasteur: Advisor/Consultant **Mary A. Staat, MD, MPH**, CDC: Grant/Research Support|Cepheid: Grant/Research Support|Merck: Grant/Research Support|NIH: Grant/Research Support|Pfizer: Grant/Research Support|Up-To-Date: Honoraria **John V. Williams, MD**, Merck: Grant/Research Support|Quidel: Board Member **Marian G. Michaels, MD, MPH**, Merck: Grant/Research Support|Viracor: Grant/Research Support

